# Tumour growth kinetics assessment: added value to RECIST in cancer patients treated with molecularly targeted agents

**DOI:** 10.1038/bjc.2012.10

**Published:** 2012-01-26

**Authors:** C Le Tourneau, V Servois, V Diéras, L Ollivier, P Tresca, X Paoletti

**Affiliations:** 1 Department of Medical Oncology, Institut Curie, 26, rue d’Ulm, 75248 Paris Cedex 05, France; 2 Department of Imaging, Institut Curie, Paris, France; 3 Department of Biostatistics, Institut Curie, Paris, France; 4 INSERM U900, Paris, France

**Keywords:** methodology, molecularly targeted agents, oncology trials, RECIST, response assessment, tumour growth kinetics

## Abstract

**Background:**

Treatment effect is categorised into four classes by RECIST based on the evolution of the size of target lesions and the occurrence of new lesions, irrespective of tumour growth kinetics before treatment. This study aimed at evaluating the added value of tumour growth kinetics assessment to RECIST in patients treated with molecularly targeted agents (MTAs).

**Methods:**

On-study imaging, along with pre-baseline imaging, of patients treated with MTA(s) in clinical trials at Institut Curie were centrally reviewed. The tumour growth ratio (TGr), defined as the ratio of the slope of tumour growth before treatment and the slope of tumour growth on treatment between the nadir and disease progression, was calculated for each patient.

**Results:**

A total of 50 patients included in 18 trials were eligible for the study. Among the 44 patients who withdrew from the study because of disease progression according to the investigators’ assessment, 18 patients (41%) had a TGr <0.9. Among these 18 patients, 5 had disease progression according to RECIST 1.1 based on our retrospective reassessment of on-study imaging and occurrence of no new lesion during study treatment.

**Conclusion:**

Our preliminary results suggest that a substantial proportion of patients treated with MTAs have discontinued treatment although being potentially benefitted from them.

Cytotoxic agents used in the metastatic setting aim at prolonging survival and/or improving the quality of life of cancer patients. In these situations, patients are usually treated until disease progression. Tumour shrinkage has classically been considered to reflect the efficacy of cytotoxic agents early, given their direct action on the cell division machinery. End points based on the size of target lesions, such as the WHO criteria and RECIST, have been developed in order to standardise the evaluation of anticancer agents (Eisenhauer *et al*, 2009). Patients with advanced disease who fail standard therapy often have rapid disease progression leading to death. However, the velocity of tumour progression might be quite heterogeneous not only across tumour types but also across patients suffering from the same tumour type, owing to different tumoural biological characteristics.

As opposed to cytotoxic agents, molecularly targeted agents (MTAs) do not always induce tumour shrinkage, but sometimes only tumour stabilisation (Llovet *et al*, 2008), which has led to the use alternate end points such as clinical benefit (defined as the addition of objective response and tumour stabilisation) or progression-free survival (PFS). However, criteria such as RECIST reduce the information by classifying treatment effect into only four categories (complete response, partial response, stable disease and progressive disease). In addition, these criteria are defined irrespective of tumour growth kinetics before treatment, and might therefore not be relevant in slow-growing diseases and for the agents that induce only tumour stabilisation.

New designs have been proposed to circumvent this caveat, including the randomised discontinuation trial or the use of PFS ratio (Ratain *et al*, 2006; Buyse *et al*, 2011). Although these latter designs take tumour growth kinetics on- and off-treatment into account for assessing efficacy, they are still based on RECIST that classify treatment effect into the only four categories described above.

We sought to evaluate whether the evaluation of tumour growth kinetics before and during treatment would add value to RECIST in patients treated with MTAs in order to determine whether some patients, in the absence of therapeutic alternative, have discontinued therapy early because of disease progression although tumour growth kinetics had been slown down.

## Patients and Methods

### Trial and patient selection

The study was approved by the Scientific Review Board of the Institut Curie. All patients with measurable lesions treated in cancer clinical trials of MTAs administered as single agents or in combination at Institut Curie between January 2005 and March 2010 were included. MTAs were defined as agents specifically modulating pathways different from those triggered by cytotoxic agents, including DNA, tubulin or the cell division machinery (Le Tourneau *et al*, 2011). Trials investigating a combination of a MTA with a cytotoxic agent or with radiotherapy were excluded. Patients with non-measurable disease, lacking pre-baseline imaging or evaluated with different imaging techniques at different time points were also excluded from the study.

### Data extraction

For all patients, the following information were recorded: age at diagnosis, gender, tumour type, number of previous lines of systemic therapy, type of last treatment administered, reason of being taken off study, best response reported in the chart, dates of pre-baseline imaging as well as on-study imaging.

All imaging were centrally and non-blindly reviewed by two senior radiologists who performed all tumour measurements on pre-baseline imaging, as well as on-study imaging. For each patient, target lesions were reassessed using RECIST 1.1 (Eisenhauer *et al*, 2009). Occurrence of new lesions during the course of the trial was also recorded.

### Statistical analysis

**Figure 1:**
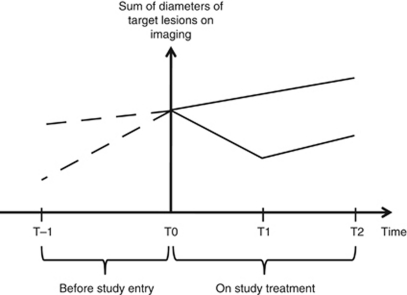
Assessment of tumour growth kinetics based on pre-study and on-study imaging measurements. Full line down=imaging measurements of a patient having an initial tumour shrinkage. Full line up=imaging measurements of a patient having a progressing disease. T=time point.

The sum of the diameters of target lesions (the smallest one for lymph nodes as per RECIST 1.1) was calculated for each patient's imaging. Let's denote *T*_pb_ and *S*_pb_, *T*_0_ and *S*_0_, *T*_N_ and *S*_N_, and *T*_eot_ and *S*_eot_, as the times of imaging and the sums of the target lesions at pre-baseline imaging, at baseline, at the nadir of response and at last study imaging, respectively. For patients not experiencing any tumour shrinkage, the nadir is considered to be the baseline. Tumour growth kinetics before the study treatment was evaluated for each patient by calculating the following slope: (*S*_0_−*S*_pb_)/(*T*_0_−*T*_pb_). Tumour growth kinetics was also evaluated while on study treatment by calculating the following slope: (*S*_eot_−*S*_N_)/(*T*_eot_−*T*_N_) (Figure 1). The ratio of these two values was named the tumour growth ratio (TGr). Following classes were used to report the TGr: <0.7, 0.7–0.9, 0.9–1.1 and ⩾1.1. As an example, a TGr of 0.8 represents a 20% slow-down of tumour growth kinetics during study treatment as compared with before study treatment.

Only the patients who received at least one dose of treatment and went off-study owing to disease progression were evaluable for the determination of the TGr. The TGr was determined using two different patient populations: (1) in all patients taken off study based on the investigators’ assessment, and (2) in the subgroup of patients with disease progression based on our retrospective reassessment of disease progression according to RECIST 1.1 and who had no new lesion during the study treatment.

Analyses were performed using the SAS software, version 9.2 (SAS Institute, Cary, NC, USA).

## Results

### Patient and trial characteristics

Among the 107 patients screened, 50 patients from 18 different trials were eligible for the study. Reasons for ineligibility included the lack of pre-baseline imaging (26 patients), non-measurable disease (12 patients), early study withdrawal because of rapid disease progression (9 patients), different imaging techniques used (9 patients) and early study withdrawal because of toxicity before any tumour evaluation (1 patient). Patient and trial characteristics are described in Tables 1 and 2, respectively. Median time between initial diagnosis and the occurrence of metastatic disease was 48 months (range: 1–484).

**Figure 2:**
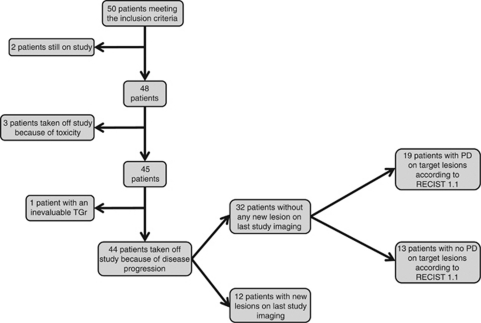
Retrospective reassessment of on-study imaging according to RECIST 1.1. PD=progressive disease; TGr=tumour growth ratio.

Of the 50 eligible patients, 48 were off-study at the time of analysis, whereas the 2 remaining patients were still on treatment. Best overall response reported in the study charts for these 48 patients was partial response in 2 patients (4%), stable disease in 18 patients (38%) and progressive disease in 28 patients (58%). The reasons of study withdrawal based on study charts information were disease progression in 45 patients (94%) and toxicity in 3 patients (6%) (Figure 2).

### Retrospective imaging reassessment

A total of 221 imaging exams were retrospectively reviewed. Median number of imaging exams per patient was 4 (range: 3–12). Median time between the pre-baseline and the baseline imaging was 10 weeks (range: 2–28).

Among the 45 patients who went off-study because of disease progression, 1 patient was not evaluable for the determination of the TGr, as the nadir occurred on last imaging assessment (concomitantly with a clinical progression). Among the remaining 44 patients, 12 patients (27%) had new lesion on last study imaging whereas 32 patients (73%) had no new lesions on last study imaging (Figure 2).

### Tumour growth ratio

Among the 44 patients eligible for the calculation of the TGr, 18 patients (41%) had a TGr <0.9, including 16 patients (36%) with a TGr <0.7. When restricted to the 32 patients with no new lesion during the study treatment, 15 patients (47%) had a TGr <0.9, including 10 patients (31%) with a TGr <0.7. Among the 12 patients who stopped treatment because of the occurrence of new lesions, 3 patients (25%) had a TGr <0.9.

**Figure 3:**
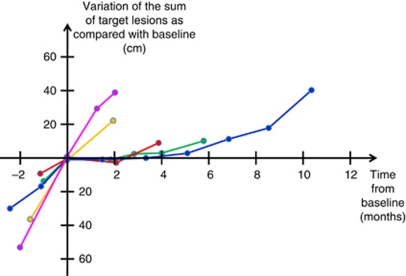
Sum of target lesions over time in the five patients with a TGr <0.9 who were taken off study because of progressive disease according to RECIST 1.1 and who had no new lesion during treatment.

Among the 19 patients who went off-study because of progressive disease on target lesions according to RECIST 1.1 based on our retrospective imaging reassessment and who had no new lesion during study treatment, 5 patients (26%) had a TGr <0.9 (Figure 3). Tumour types of these five latter patients included breast adenocarcinoma (three patients), melanoma (one patient) and sarcoma (one patient).

## Discussion

Our study shows that as many as 18 patients out of the 44 patients who withdrew from the study because of disease progression had a TGr <0.9. Among these 18 patients, 5 had a progressive disease according to RECIST 1.1 based on our retrospective reassessment of on-study imaging as well as no new lesion during treatment. This means that 5 patients out of the initial set of the 44 patients went off-study based on RECIST 1.1, although the slope of tumour growth had been broken following study treatment by 10% or more. Although the retrospective nature of this work along with the absence of randomisation preclude to draw robust conclusions, our preliminary results, therefore, suggest that a substantial proportion of patients treated with MTAs discontinued treatment whilst the tumour growth under treatment was still slower than before treatment. Whether slowing down tumour progression would translate into survival prolongation and/or quality of life improvement remains to be determined. However, it might serve as a sign of activity in early phase clinical trials.

Our study is original in two ways. The first one is the use of a continuous variable to assess the treatment effect instead of the four categories proposed in RECIST. The limitation of using binary end points for detecting and/or quantifying the effects is obvious for MTAs that might not induce any tumour shrinkage although being able to prolong survival (Llovet *et al*, 2008). Worse, some classes of agents such as antiangiogenic agents and immunotherapeutics might induce a transient tumour growth that should not be interpreted as a disease progression (Crabb *et al*, 2009; Hales *et al*, 2010).

The second original point of our study is the use of pre-treatment information to assess the treatment effect. Several efficacy end points have previously been reported in the literature that used pre-treatment information (Von Hoff, 1998; Mick *et al*, 2000; Zalcberg *et al*, 2005; Debiec-Rychter *et al*, 2006; Von Hoff *et al*, 2010). Von Hoff introduced more than a decade ago the ‘growth modulation index’, which is a ratio of times to progression (TTP) (Von Hoff, 1998; Mick *et al*, 2000). This ratio has been used as an end point in some trials in which the treatment dose could be increased at the time of progression (Zalcberg *et al*, 2005; Debiec-Rychter *et al*, 2006). In these trials, a dose increase was considered efficient if the TTP ratio exceeded a prespecified threshold (namely 1.25 and 1.33, respectively). More recently, Von Hoff *et al* (2010) used patients as their own control to assess the efficacy of a treatment based on molecular profiling by calculating for each patient the ratio of the PFS on treatment and the PFS on the previous treatment. Therapy based on molecular profiling was considered to be effective for a given patient if the ratio exceeded 1.3. However, these ‘growth modulation indexes’ are valid only if the underlying assumption that there is a strong correlation between the two TTP/PFS is verified. In case of the absence of correlation, this ratio gets non informative. Buyse *et al* (2011) have shown from data obtained in a prospective trial in metastatic colorectal cancer that this latter assumption might not always be verified.

To our knowledge, only one study beside our study retrospectively evaluated tumour growth kinetics before and during treatment for the evaluation of treatment efficacy (Gomez-Roca *et al*, 2011). This study differed from our study by the following points: (1) only patients participating in phase I trials were included, and (2) treatments included not only MTAs but also cytotoxic agents (alone or in combination). The authors reported a significant association between a TTP >12 weeks and a decrease in the tumour growth rate, defined as a change in tumour volume per month, during the study treatment.

Two aspects of the results deserve further attention. First, a substantial proportion of patients (25%) with new lesions occurring during the treatment had a TGr <0.9. One may ask whether the occurrence of new lesions should necessarily lead to treatment discontinuation in all cases. In addition, the occurrence of new lesions might sometimes be transient, reflecting a flare-up phenomenon, such as bone lesions in castrate-resistant prostate cancer or in metastatic gastric cancer (Amoroso *et al*, 2007; Messiou *et al*, 2011). Finally, it is highly probable that different sites of new lesions may have different impacts on prognosis, such as bone *vs* visceral lesions (Imkampe *et al*, 2007). Similarly, multiple new lesions probably do not carry the same information as an isolated one when other lesions are clearly reduced or stabilised. Second, 30% of the 44 patients who had disease progression according to the investigators assessment went off-study without progressive disease according to RECIST 1.1. Explanations for this include differences between the investigators’ assessment according to RECIST 1.0 or 1.1 (depending on the studies) and our reassessment according to RECIST 1.1, and withdrawal from study because of clinical progression.

Our study has several limitations: (1) the small size that only allows generating hypotheses, (2) the inclusion of a majority of patients with breast cancer and sarcoma, (3) data obtained from a single institution, and (4) the retrospective nature of this study. However, our results suggest that the evaluation of tumour growth kinetics before and on treatment adds value to RECIST in patients treated with MTAs.

The TGr presents several advantages. First, it displays a higher statistical power to detect modifications than RECIST. Second, it allows standardising on inter-patients’ variability, each patient being its own control. Third, hints of antitumour activity can thereby be detected in heavily pretreated patients whose tumours are expected to be less sensitive to new treatments. In these patients, one might want to continue the treatment even in case of disease progression according to RECIST if tumour growth is being slowed down by the treatment. It remains to be demonstrated prospectively whether this approach would translate into improved survival and/or quality of life.

## Figures and Tables

**Table 1:** Patient characteristics

	***N***	**%**	**Median**	**Range**
Age at study entry			51	(15–76)
				
*Gender*				
Male	9	18		
Female	41	82		
				
*Tumour type*				
Breast adenocarcinoma	20	40		
Sarcoma	19	38		
Melanoma	6	12		
Ovarian adenocarcinoma	2	4		
Colorectal adenocarcinoma	1	2		
Pancreatic adenocarcinoma	1	2		
Non-small cell lung cancer	1	2		
				
Prior lines of chemotherapy/MTA			3	(0–8)
				
*Type of last treatment received*				
Chemotherapy±MTA	32	64		
MTA	5	10		
Hormone therapy	3	6		
None	10	20		

Abbreviation: MTA=molecularly targeted agent.

**Table 2:** Trial characteristics

	**No. of trials**	**%**	**No. of patients**	**%**
*Number of MTAs*				
Single agent	13	72	42	84
Combination	5	28	8	16
				
*Trial phase*				
Phase I	3	17	16	32
Phase I/II	2	11	2	4
Phase II	12	67	28	56
Phase III	1	6	4	8
				
*Targets of MTAs* a				
HER-2	5	22	7	13
VEGFR	4	17	7	13
EGFR/HER-2	4	17	11	21
IGF-1R	3	13	7	13
EGFR	1	4	1	2
mTOR	1	4	1	2
CDK	1	4	3	6
SRC	1	4	2	4
HDAC	1	4	1	2
PKC	1	4	4	8
MEK	1	4	9	17

Abbreviation: MTA=molecularly targeted agent.

a The number of MTAs is superior to the number of trials as five of the trials are combination trials.
